# Evaluation of maslinic acid with whole-body vibration training in elderly women with knee osteoarthritis

**DOI:** 10.1371/journal.pone.0194572

**Published:** 2018-03-20

**Authors:** Jieun Yoon, Akihiro Kanamori, Keisuke Fujii, Hiroko Isoda, Tomohiro Okura

**Affiliations:** 1 Faculty of Health and Sport Science, University of Tsukuba, Tsukuba, Japan; 2 Faculty of Medicine, University of Tsukuba, Tsukuba, Japan; 3 Doctoral Program in Physical Education, Health and Sport Science, University of Tsukuba, Tsukuba, Japan; 4 Faculty of Life and Environmental Science, University of Tsukuba, Tsukuba, Japan; 5 Alliance for Research on North Africa (ARENA), University of Tsukuba, Tsukuba, Japan; University of L’Aquila, ITALY

## Abstract

**Purpose:**

Maslinic acid (MA) is a component derived from a natural olive-based extract known to have pharmacological functions that include suppressing inflammation. This study examined how MA, in conjunction with whole-body vibration training (WBVT), can improve knee and muscle function in elderly women with knee osteoarthritis (OA).

**Methods:**

The study was a double-blinded, placebo-controlled, randomized intervention study that enrolled individuals with knee pain. Participants were 26 females aged 65–85 years with knee OA. They performed WBVT and ingested either 16.7 mg of MA or a placebo daily for 20 weeks. We compared the effect of WBVT with placebo (WBVT/P) and WBVT with MA (WBVT/MA) in participants with various degrees of knee OA (Kellgren and Lawrence (K-L) grade) using the Japanese Orthopaedic Association (JOA) score and isokinetic dynamometer measurements to evaluate knee and muscle function with two-way ANOVA.

**Results:**

Based on the results of two-way ANOVA analysis of muscle function measurements, there was significant interaction (time × group) (*P* = 0.03) in the “isokinetic extension peak torque” domain for severe OA (K-L grade ≥ 3). The simple main effect of time in the WBVT/MA group (*P* = 0.04) contributed to this interaction. The JOA score for WBVT/MA supported the main effect of group as having a significant correlation in the “pain on walking” (*P* = 0.04) and “range of motion” (*P* < 0.01) domains. Participants with severe knee OA in the WBVT/MA group improved in these domains, whereas the WBVT/P group had few positive results.

**Conclusions:**

Participants with severe OA who ingested MA in conjunction with WBVT improved their knee and muscle function. This study suggests that ingesting the anti-inflammatory supplement MA while participating in WBVT, elderly women can reduce knee OA and improve their knee muscle strength.

## Introduction

Knee osteoarthritis (OA) is one of the most common knee joint diseases in the elderly with 25.3 million elderly people affected in Japan alone [1]. Japanese women have traditionally sat seiza style in which they kneel on their lower legs while resting their buttocks on their heels [2]. Thus, knee OA in Japanese elderly women may be more severe than in their Western counterparts. Knee OA involves synovial inflammation and cartilage degradation, and patients with a severe Kellgren-Lawrence (K-L) grade (K-L ≥ 3) have synovitis or joint effusion [3]. Furthermore, severe knee OA can weaken the surrounding muscles [4]. In particular, reduced quadriceps strength is both a risk factor and a consequence of knee OA [5, 6]. Therefore, many patients with knee OA have difficulty with their regular routines and daily movements such as walking or standing, and in severe cases, they require nursing home care.

The Osteoarthritis Research Society International (ORSI) currently recommends a combination of pharmacological and nonpharmacological treatments as part of nonsurgical management for patients with knee OA [7]. However, there is a greater risk of adverse drug events in elderly people, and they may experience side effects such as gastrointestinal problems, kidney damage or even kidney failure with the administration of medications such as painkillers and anti-inflammatory drugs [8]. Consequently, the ideal treatment for elderly patients with knee OA may be a combination of effective and natural supplements instead of anti-inflammatory drugs.

Maslinic acid (MA) is a pentacyclic triterpene derived from olives that has produced no pharmacological problems for elderly people. Its pharmacological functions include anti-inflammatory, anti-tumor and anti-oxidative properties, among others [9, 10]. Thus, the anti-inflammatory activity of MA may relieve pain in patients with knee OA.

Resistance exercise is a non-pharmacological treatment that can reduce pain and improve knee function. Maintaining muscle strength is extremely important for patients with knee OA [11]. Whole-body vibration training (WBVT), a type of neuromuscular training, is an efficient resistance exercise method [12]. It has become a popular alternative exercise in hospitals and fitness clubs because WBVT is typically not burdensome for injured patients or the elderly. In WBVT, the participant stands on a platform that generates vertical sinusoidal vibrations. Previous studies have shown that WBVT can improve knee pain, knee function and muscle function in elderly people with knee OA [13–16].

However, in our examination of the published literature, we found little information on enhancing the effects of anti-inflammatory agents, such as supplements, with resistance exercise such as WBVT. Therefore, the purpose of this study is to evaluate the efficacy of WBVT in conjunction with MA supplementation for improving elderly women’s knee and muscle function. In our double-blind study, we compared parameters of knee OA in patients before and after they participated in a WBVT program while receiving either MA (WBVT/MA group) or a placebo (WBVT/P group). We also examined whether the degree of OA affects a patient’s response to MA during WBVT by comparing the results of participants who had K-L grades ≤ 2 with those who had K-L grades ≥ 3. We investigated whether MA’s anti-inflammatory properties could enhance the effects of WBVT for patients with knee OA.

## Materials and methods

This was a double-blinded, placebo-controlled, randomized intervention study in which we enrolled individuals with knee OA for 20 weeks. A computer randomization list provided by a research randomizer program (http://www.randomizer.org) assigned participants to either the WBVT/MA or the WBVT/P group. We gathered all test measurements before and after the 20-week training period.

### Participants

The study consisted of a resistance exercise (WBVT) and ingestion of MA or placebo over 20 weeks. We recruited women from nearby residential areas during the two-week period prior to the study through an advertisement in a local newspaper. We used the following criteria for selecting study participants from the women who responded: participants must (1) be 65 years or older, (2) have pain in one or both knees, and (3) not have engaged in organized regular physical exercise, sports or strength training. Furthermore, we did not include participants with various health issues such as those with acute disease or severe diabetes, we also excluded those who were currently engaged in training sessions. We selected 26 of the 97 willing participants, and their ages ranged from 65–85 years old with a mean of 70.3 years. We informed the participants not to take any anti-inflammatory or analgesic medications to relieve pain during the 20-week study. All participants agreed to the study rules.

The Ethics Committee of the University of Tsukuba approved this study (No., Tai 26–24). All participants provided written informed consent for their participation in the study.

### Baseline characteristics

Table 1 shows participants’ baseline characteristics of age, height, weight, body mass index (BMI), the knee OA grade, duration of knee pain and/or stiffness, existence of any chronic diseases and radiological severity of OA.

**Table 1:** Participants’ characteristics.

		WBVT/MA (n = 11)		WBVT/P (n = 15)		*P*-value
		Mean ± SD	Range	Mean ± SD	Range	
**Age**	**years**	69.5 ± 2.9	65–73	71.0 ± 5.2	65–85	0.40
**Height**	**cm**	152.8 ± 4.8	142–159	151.7 ± 5.6	142–159	0.62
**Body weight**	**kg**	53.4 ± 7.7	38–65	56.0 ± 8.7	43–72	0.43
**Body mass index**	**kg/m**^**2**^	22.8 ± 2.9	17–27	24.4 ± 4.1	18–33	0.29
**K-L grade ≤ 2**		Knees = 15 (63.6%)		Knees = 12 (40.0%)		0.14^a^
**K-L grade ≥ 3**		Knees = 7 (36.4%)		Knees = 18 (60.0%)		

SD: standard deviation, *P*-value: Student’s *t*-test,

^a^: chi-squared test.

WBVT/MA: whole-body vibration training + maslinic acid capsules, WBVT/P: whole-body vibration training + placebo. K-L: Kellgren and Lawrence.

### Kellgren and Lawrence (K-L) grade

Although a physician did not evaluate participants’ knees for entry into the study, all of them experienced knee pain. Radiographs for knee joint angles, femorotibial angle, anatomical lateral distal femoral angle and anatomical medial proximal tibial angle were measured at Tsukuba Memorial Hospital, Tsukuba, Japan. The radiographs of participants’ knees were assigned K-L grades (K-L grade ≤ 2 or K-L grade ≥ 3) in order to rank knee OA severity. Generally, radiologic knee OA is defined as having at least a K-L grade of 1 or more [17].

### Maslinic acid (MA) and placebo

**Fig 1:**
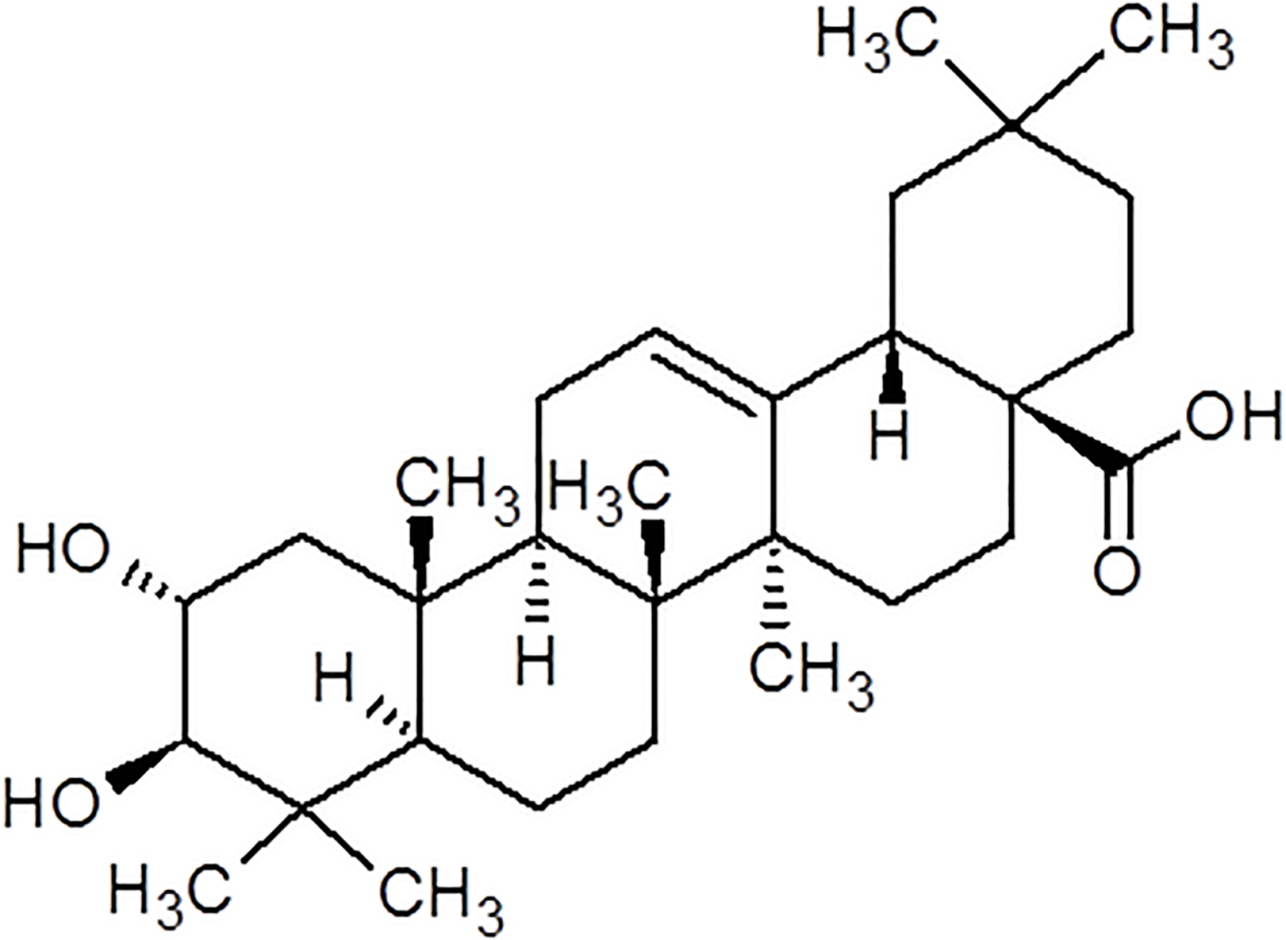
Structure of maslinic acid ((2α,3β)-2,3-Dihydroxyolean-12-en-28-oic acid).

MA (2α, 3β-dihydroxyolean-12-en-28-oic acid) is extracted from the fruit of olives (Fig 1). The MA product is a non-pharmacological (natural extract) health food that was approved in Japan prior to our study as a safe to use supplement. Nippon Flour Mills Co, Ltd., Tokyo, Japan provided the MA and placebo capsules for this double-blind study. The MA capsules were composed of approximately 10.7% MA, 67% gamma-cyclodextrin, 22.8% protein, 1.5% fat, 2.5% ash, 3.4% moisture and other minor components. The placebo capsules contained cornstarch instead of olive fruit extract and were prepared to match the shape and color of the MA capsules. There were 11 participants in the WBVT/MA group and 15 participants in the WBVT/P group. The staff checked the records once a month and left comments to motivate the participants. The participants ingested three capsules of either MA or placebo once daily at breakfast for 20 weeks.

### Whole-body vibration training (WBVT)

We designed the WBVT program mainly to strengthen the hamstrings, calf muscles, quadriceps and surrounding muscles using squats and lunge exercises (Table 2). The program consisted of squats (R1), calves (R2), sit-ups (R3) and up and down (R4). Participants performed all exercises on vertical vibration machines (POWER PLATE pro5, POWER PLATE International, London, UK).

**Table 2:** Characteristics of the whole-body vibration training program.

Week	Exercise category	Exercise: Muscles targeted	Time(s/set)	Number of sets
**1 → 4**	**Warm-up(6 exercises)**	Hamstring stretch (W1): Hamstring	30	1 (each leg)
		Calf stretch (W2): Calf muscles	30	1 (each leg)
		Side stretch (W3): Obliquus externus abdominis	30	1 (each side)
		Quadriceps stretch (W4): Quadriceps	30	1 (each leg)
		Back relaxer (W5): Latissimus dorsi	30	1
		Hip stretch (W6): Gluteus maximus	30	1 (each side)
	**Resistance training (4 exercises)**	Squat (R4): Quadriceps, Gluteus maximus, Hamstring	30	1 (1st week) → 2 (2nd week)
		Calves (R4): Calf muscles	30	1 → 2
		Sit-up (R4): Rectus abdominis	30	1 → 2
		Up and down (R4): Biceps (Triceps) brachii, Trapezius, Quadriceps	30	1 (each leg)
				→ 2
	**Cool-down (3 exercises)**	Calf massage (C4): Calf muscles	60	1
		Hamstring massage (C4): Hamstring	60	1
		Quadriceps massage (C4): Quadriceps	60	1
		Back massage (C4): Latissimus dorsi	60	1
**5 → 10**	**Warm-up**	(The same 6 exercises as 1st through 4th weeks)		
	**Resistance training (5 exercises)**	(The same 4 exercises as the 4th week): Quadriceps + Pelvic bridge (R5): Gluteus maximus, Hamstring	30	1 (3rd week) → 2 (5th week)
	**Cool-down**	(The same 4 exercises as 1st through 4th weeks)		
**11→ 20**	**Warm-up**	(The same 6 exercises as 1st through 4th weeks)		
	**Resistance training (6 exercises)**	(The same 5 exercises as the 5th week): Quadriceps + Front lunge (R6): Gluteus maximus	30	1 (each leg) (11th week) → 2 (13th week)
	**Cool-down**	(The same 4 exercises as 1st through 4th weeks)		

### Muscle function evaluation

We used the Biodex isokinetic dynamometer (Biodex Medical Systems, Shirley, NY, USA) to test the isometric (0 deg/s) knee extension peak torque and isokinetic (60 deg/s) knee extension/flexion peak torque of both knees in each participant. Participant positioning for the knee extension and flexion trials has been described previously [18]. Participants performed maximal isometric knee extensions of 3-s duration at a knee joint angle of 120 deg (180 deg = full knee extension) [19]. They performed isokinetic knee extension/flexion trials separately three times with knee joint angle ranging from approximately 90 deg to 80 deg. For each trial, participants performed two submaximal and two maximal contractions before testing. They then performed three maximal voluntary contractions, each one separated by a 5-s pause. A test period of at least 5 min was allowed between each trial to exclude the effect of fatigue. We used the Biodex System 4 Advantage software (version 4. 26b) to calculate peak torque and recorded the highest value from the three trials. Peak torque data were normalized per kilogram of body weight (Nm/kg).

### Knee function evaluation

The Japanese Orthopaedic Association (JOA) score is a scale created by the association in 1998 to measure mobility difficulty in patients with knee OA [20]. It is based on both a subject interview and the diagnosis of an orthopedist. In the present study, we acquired JOA score measurements on both knees of the participants. Participants graded their subjective condition on a scale of 0–100 with 100 indicating “very good” in the following four areas: pain on walking, pain on ascending or descending stairs, range of motion and joint effusion. This grading system numerically evaluated difficulties in walking and climbing stairs, mobility level and joint swelling.

### Statistical analysis

We compared baseline characteristics as mean with standard error or as frequency between groups using non-paired *t*-test or chi-squared test, respectively. Two-way analysis of variance (ANOVA), was used to determine whether differences existed between values (two levels for the time factor: pre- and post-trial) for each group (group factor: WBVT/MA and WBVT/P) according to all outcome measures. When an interaction was significant, we examined the simple effect of time and group. In addition, when interaction was not significant, we tested the main effect of time and group. We calculated 95% confidence intervals for all outcome measures pre- and post-trial. The effect sizes (Cohen’s *d*) between pre- and post-trial data were determined by using the average change and excluding the pre-test standard deviation. The calculated intraclass correlation coefficient (ICC) based on pre- and post-trial data was moderate or greater (0.41~0.87). The calculated coefficient of variation based on pre-test data was 0.11 to 0.36. Effect size (*d*) standards were 0.2 = small, 0.5 = moderate and 0.8 = large. IBM SPSS Statistics 22 software for Windows (IBM, Armonk, NY) performed all statistical processing. Statistical significance was set at *P* < 0.05.

## Results

### Baseline characteristics and the trial of MA and placebo

The 26 participants’ ages ranged from 65–85 years with a mean of 70.3 years, and their mean body mass index (BMI) was 23.7 kg/m^2^ with a range of 16.5–32.7 kg/m^2^ (Table 1). The Japanese Ministry of Health, Labour and Welfare (2013) lists the average BMI at 22.6 kg/m^2^ between 60–69 years and 23.0 kg/m^2^ at 70 years and older. Therefore, participants’ BMIs are typical of this Japanese age population.

We determined the Kellgren and Lawrence (K-L) grade for both knees of the participants through radiographs. From these results, we found that 51.9% (27 knees) of participants had K-L grade ≤ 2 and 48.1% (25 knees) had K-L grade ≥ 3. Participants’ reported two patterns of knee pain. One in which the participants were having knee pain on both sides of a knee, one in which the knee pain was only on one side of the knee. The number of knees with pain that had a K-L grade ≤ 2 in the WBVT/MA group was 15 and in the WBVT/P group it was 12. The number of knees with pain that had a K-L grade ≥ 3 in the WBVT/MA group was 7 and in the WBVT/P group it was 18. There were no significant differences (*P* < 0.05) between the WBVT/MA and WBVT/P groups. All participants were diagnosed with knee OA. Participants consumed either three 50 mg MA capsules (16.7 mg of MA) or three placebo capsules at breakfast, depending on their assigned group. This continued over the 20-week program. Therefore, the participants in the WBVT/MA group consumed 2338 mg over the entire trial period of 20 weeks.

### Performance of WBVT program

Table 2 shows the progression of exercises in the WBVT program for this study. We calculated the training session attendance rates for each group. There were 440 participant-training hours available for the WBVT/MA group (11 participants × 40 sessions) and 600 participant-training hours available for the WBVT/P group (15 participants × 40 sessions). Members of the WBVT/MA group attended 422 hours, which was a 95.9% attendance rate, and members of the WBVT/P group attended 581 hours for a 96.8% attendance rate.

Depending on the results of participants’ first four weeks in the program, we added other exercises to strengthen knee muscles, such as the pelvic bridge (R5), during weeks five to ten. If the participants progressed sufficiently during the first ten weeks of the program, we challenged them during the last ten weeks of the program with more difficult strengthening exercises such as the front lunge (R6). Although all participants entered the WBVT program with knee pain, they all successfully progressed through our entire WBVT program.

### Muscle function

Table 3 indicates the pre- and post-trial muscle function measurement outcomes and results of the two-way ANOVA. There was significant time × group interaction in the “isokinetic extension peak torque” domain (*P* = 0.03) for participants with K-L ≥ 3. The simple main effect of time in the WBVT/MA group contributed to this result (*P* = 0.04). In contrast, the other domains showed no significant time × group interactions.

**Table 3:** Muscle function by group at baseline and follow-up.

	Week	Mean SD	95% CI	Effect size Cohen’s *d*	Mean SD	95% CI	Effect size Cohen’s *d*	Interaction	Main effect	Simple main effect (time)
**Kellgren & Lawrence Grade ≤ 2**		**WBVT/MA (knees = 15)**			**WBVT/P (knees = 12)**					
**Isometric extension peak torque, Nm/kg**	0	1.68 ± 0.25	(1.53–1.83)	0.92	1.54 ± 0.47	(1.24–1.84)	0.38	0.62	Time *P* < 0.01	
	21	1.95 ± 0.33	(1.75–2.15)		1.73 ± 0.52	(1.40–2.08)			Group *P* = 0.22	
**Isokinetic extension peak torque, Nm/kg**	0	1.17 ± 0.33	(0.97–1.37)	0.37	1.06 ± 0.42	(0.79–1.33)	0.52	0.70	Time *P* < 0.01	
	21	1.31 ± 0.42	(1.06–1.57)		1.25 ± 0.30	(1.06–1.44)			Group *P* = 0.53	
**Isokinetic flexion peak torque, Nm/kg**	0	0.79 ± 0.20	(0.67–0.91)	0.90	0.78 ± 0.18	(0.66–0.90)	0.66	0.77	Time *P* = 0.01	
	21	0.97 ± 0.18	(0.86–1.08)		1.00 ± 0.44	(0.72–1.28)			Group *P* = 0.91	
**Kellgren & Lawrence Grade ≥ 3**		**WBVT/MA (knees = 7)**			**WBVT/MA (knees = 18)**					
**Isometric extension peak torque, Nm/kg**	0	1.42 ± 0.35	(1.15–1.69)	0.43	1.19 ± 0.28	(1.06–1.33)	-0.04	0.10	Time *P* = 0.15	
	21	1.58 ± 0.40	(1.28–1.89)		1.18 ± 0.30	(1.03–1.33)			Group *P* < 0.01	
**Isokinetic extension peak torque, Nm/kg**	0	0.82 ± 0.33	(0.56–1.07)	0.59	0.90 ± 0.32	(0.74–1.05)	-0.24	0.03		WBVT/MA *P* = 0.04
	21	1.01 ± 0.31	(0.82–1.29)		0.84 ± 0.18	(0.75–0.92)				WBVT/P *P* = 0.43
**Isokinetic flexion peak torque, Nm/kg**	0	0.64 ± 0.13	(0.54–0.74)	0.98	0.71 ± 0.28	(0.57–0.85)	0.44	0.59	Time *P* = 0.04	
	21	0.80 ± 0.19	(0.65–0.95)		0.81 ± 0.15	(0.73–0.88)			Group *P* = 0.53	

Cohen’s *d*: |0.2 ≤ *d* < 0.5| = small, |0.5 ≤ *d* < 0.8| = moderate, |0.8 ≤ *d*| = large.

*P* < 0.05, CI: confidence interval.

WBVT/MA: whole-body vibration training + maslinic acid capsules, WBVT/P: whole-body vibration training + placebo.

For the main effect of time, there were significant results in all domains for participants with K-L ≤ 2 (*P* < 0.01 or *P* = 0.01) and in the “isokinetic flexion peak torque” for participants with K-L ≥ 3 (*P* = 0.04). The “isometric extension peak torque” for participants with K-L ≥ 3 had a significant result only in the main effect for that group (*P* < 0.01).

For knee strength in the WBVT/P group, participants with K-L ≤ 2 knees had small or moderate effect sizes (*d* = 0.38–0.66) for all domains, and for K-L ≥ 3 knees, the effect size for the “isokinetic flexion peak torque” was small (*d* = 0.44). Furthermore, the effect size for the “isokinetic and isometric extension peak torque” indicated a worsening knee for the WBVT/P participants with K-L ≥ 3 (*d* = -0.24 –-0.04).

On the other hand, the effect sizes of all domains in the WBVT/MA group were higher than the effect sizes in the WBVT/P group. In particular, “isometric extension peak torque” (*d* = 0.92) and “isokinetic flexion peak torque” (*d* = 0.90) for participants with K-L ≤ 2, and “isokinetic flexion peak torque” (*d* = 0.98) for those with K-L ≥ 3 were remarkably higher in the WBVT/MA group than in the WBVT/P group.

### Knee function

Table 4 shows the pre- and post-trial means and 95% confidence intervals for knee function measurements and results of the two-way ANOVA. There was no significant time × group interaction in knee function (*P* = 0.24–0.84). However, we found a significant main effect of time in “pain on ascending or descending stairs” for participants with K-L ≤ 2 (*P* < 0.01). In the K-L ≥ 3 group, the main effect of group in the “pain on walking” (*P* = 0.04) and “range of motion” (*P* < 0.01) domains was significant.

**Table 4:** Japanese Orthopaedic Association score by group at baseline and follow-up.

	Week	Mean SD	95% CI	Effect size Cohen’s *d*	Mean SD	95% CI	Effect size Cohen’s *d*	Interaction	Main effect
**Kellgren & Lawrence Grade ≤ 2**		**WBVT/MA (knees = 15)**			**WBVT/P (knees = 12)**				
**Pain on walking, score**	0	28.8 ± 2.2	(27.5–30.2)	0.43	29.6 ± 1.4	(28.7–30.5)	0.41	0.61	Time *P* = 0.09
	21	29.6 ± 1.4	(28.8–29.9)		30.0 ± 0.0	–			Group *P* = 0.27
**Pain on ascending or descending stairs, score**	0	21.2 ± 4.2	(18.4–23.7)	0.62	20.8 ± 1.9	(19.6–22.1)	1.13	0.84	Time *P* < 0.01
	21	23.5 ± 3.2	(21.6–25.4)		23.3 ± 2.5	(21.8–24.9)			Group *P* = 0.84
**Range of motion, score**	0	31.9 ± 4.8	(29.0–34.8)	0.21	33.3 ± 2.5	(31.8–34.9)	0.18	0.75	Time *P* = 0.28
	21	32.7 ± 2.6	(31.1–34.3)		33.8 ± 2.3	(32.3–35.2)			Group *P* = 0.31
**Joint effusion, score**	0	9.6 ± 1.4	(8.8–10.5)	–	10.0 ± 0.0	–	–	–	–
	21	9.6 ± 1.4	(8.8–10.5)		10.0 ± 0.0	–			–
**Kellgren & Lawrence Grade ≥ 3**		**WBVT/MA (knees = 7)**			**WBVT/P (knees = 18)**				
**Pain on walking, score**	0	28.3 ± 2.5	(26.4–30.3)	–	26.9 ± 4.2	(24.8–29.1)	-0.57	0.26	Time *P* = 0.28
	21	28.3 ± 2.5	(26.4–30.3)		24.2 ± 5.5	(21.4–26.9)			Group *P* = 0.04
**Pain on ascending or descending stairs, score**	0	16.7 ± 7.9	(10.6–22.7)	0.39	16.7 ± 6.4	(13.5–19.9)	-0.22	0.24	Time *P* = 0.69
	21	19.4 ± 5.8	(15.0–23.9)		15.3 ± 6.5	(12.03–18.5)			Group *P* = 0.33
**Range of motion, score**	0	32.2 ± 2.6	(30.2–34.2)	0.71	28.1 ± 4.6	(25.8–30.3)	0.12	0.39	Time *P* = 0.09
	21	33.9 ± 2.2	(32.2–35.6)		28.6 ± 4.8	(26.2–31.0)			Group *P* < 0.01
**Joint effusion, score**	0	9.4 ± 1.7	(8.2–10.7)	0.50	8.6 ± 2.3	(7.5–9.8)	0.10	0.47	Time *P* = 0.81
	21	10.0 ± 0.0	–		8.3 ± 3.4	(6.6–10.0)			Group *P* = 0.15

Cohen’s *d*: |0.2 ≤ *d* < 0.5| = small, |0.5 ≤ *d* < 0.8| = moderate, |0.8 ≤ *d*| = large.

*P* < 0.05, CI: confidence interval.

WBVT/MA: whole-body vibration training + maslinic acid capsules, WBVT/P: whole-body vibration training + placebo.

The effect size (Cohen’s *d*) of “pain on ascending or descending stairs” for participants with K-L ≤ 2 was large only in the WBVT/P group (*d* = 1.13). Whereas in the WBVT/MA group, participants with K-L ≤ 2 had a moderate effect size for “pain on ascending or descending stairs” (*d* = 0.62), and participants with K-L ≥ 3 had moderate effect sizes for “range of motion” (*d* = 0.71) and “joint effusion” (*d* = 0.50). Furthermore, the effect sizes for “pain on walking” and “pain on ascending or descending stairs” of participants in the WBVT/P group with a K-L ≥ 3 score indicated worsening knee pain (*d* = -0.57, -0.22, respectively).

## Discussion

We designed this study for elderly women to participate in a resistance exercise program, such as WBVT, over 20 weeks while receiving either a MA supplement (WBVT/MA group) or a placebo (WBVT/P group). Since knee pain can worsen during cold weather, all participants started and completed the WBVT exercise program prior to the cold winter weather. Unfortunately, we only had five WBV units available for our study allowing us to include a relatively small number of participants, which was a limitation of our study.

All participants (elderly women ranging from 65–85 years) identified themselves as having knee OA: 51.9% (knees = 27) had K-L grades ≤ 2 and 48.1% (knees = 25) had K-L ≥ 3 (Table 1). According to D’Agostino et al. [3], participants with knee OA and a K-L grade ≥ 3 have synovitis or joint effusion on clinical examination and present with knee joint swelling. Their results showed a high correlation between the radiographic stage of knee OA and the inflammatory process. The inflammation correlated statistically with advanced radiographic disease (K-L grade ≥ 3 for synovitis and joint effusion). Indeed, participants with a K-L grade ≥ 3 in this study also had synovitis or joint effusion and inflammation in their knees.

Maslinic acid derived from olives has been garnering attention as a dietary supplement in Japan; it reduces inflammation and swelling [21, 22]. Fukumitsu et al. [23] reported that MA can suppress mild knee pain and improve quality of life (QoL) by promoting weight loss in the elderly. MA affects the production of tumor necrosis factor-a (TNF-a), a type of cytokine which causes rheumatism [10]. Even though MA is considered a natural and safe supplement, it can potentially reduce inflammation in patients with knee OA similar to an anti-inflammatory drug. To study MA’s ability to relieve knee arthritis in elderly women, we conducted a study with participants that had knee OA. To reduce inflammation in the osteoarthritic knees, we incorporated treatment with MA, which has no pharmacological side effects, with resistance exercise (WBVT) to improve muscle strength over a 20-week period. Our two-way ANOVA analysis of the muscle function measurements provided a quantitative evaluation of participants’ knee joints and knee muscles (Table 3).

In particular, “isokinetic extension peak torque” had a significant time × group interaction in participants with knees graded at K-L ≥ 3. This indicates that the effectiveness of WBVT with MA supplementation (WBVT/MA group) depends on the time parameter. The detailed findings support the significance of the simple main effect of time for participants with K-L ≥ 3 (*P* = 0.04). From these results, we found that WBVT led to improved muscle strength and that MA is more effective when the K-L grade is severe (K-L ≥ 3) as opposed to mild or moderate (K-L ≤ 2). Thus, it is clear from our results that the elderly participants with more serious knee OA who received MA during the 20-week exercise program (WBVT/MA) received considerable benefit from this combination.

The effect size (*d*) results also revealed that participants in the WBVT/MA group had better muscle function than those in the WBVT/P group in all domains. MA seemed to improve muscle strength as seen with isokinetic knee extension/flexion peak torque measurements. From these results, we found that WBVT led to improved muscle strength and that MA is more effective when the K-L grade is severe (K-L ≥ 3) as opposed to mild or moderate (K-L ≤ 2). This is supported by MA’s pharmacological function as an anti-inflammatory agent. Synovitis and effusion are common in painful knee OA, and the presence of synovitis and effusion are strongly related to medial hamstring function. Rutherford et al. [24] and Torry et al. [25] reported that vastus medialis and vastus lateralis activity decreased, on average, 8.5% and 50%, respectively. In general, hamstring function is related to a loss of knee extension strength [26, 27]. Furthermore, Lewek et al. [28] observed poor knee extension strength in participants with knee OA. There must be a critical interaction between dynamic and passive muscle forces to stabilize the knee joint during walking, running, jumping and the like. This is a possible explanation for the strong relationship between knee joint effusion and hamstring muscle strength.

Specifically, our WBVT program consists of hamstring stretch (W1), squat (R1), hamstring massage (C2) and pelvic bridge (R5) (Table 2) because these exercises correlate to strengthening of the hamstring muscles. Tillaar [26] also reported that WBVT, in particular, may improve flexibility of the hamstrings. In addition, Mathers et al. [27] reported that the inflammatory responses to exercise-induced muscle damage influence subsequent muscle regeneration.

The JOA scores (two-way ANOVA) showed that there was no significant time × group interaction for knee function (Table 4). However, as an individual main effect, time had a significant correlation (*P* < 0.01) with “pain on ascending or descending stairs” for participants with K-L ≤ 2. This makes sense since participants with mild knee OA increased muscle mass around their knees during the 20-week WBVT program, making it easier to ascend and descend stairs without knee pain. Furthermore, the participants with K-L grade ≤ 2 had no synovitis or joint effusion, so their knee pain was unrelated to knee inflammation.

WBVT was a useful resistance training exercise for all study participants with mild knee OA (K-L ≤ 2) regardless of the supplementation; the exercise helped them build leg muscles and improved their ability to ascend and descend stairs. This result is consistent with previous studies of WBVT [13–16]. We demonstrated that WBVT can improve knee pain in elderly people with knee OA and improve knee function in joints with mild OA.

On the other hand, for participants with severe OA (K-L ≥ 3), the main effect of group correlated significantly with “pain on walking” (*P* = 0.04) and “range of motion” (*P* < 0.01). The WBVT/MA group improved in these domains, but the WBVT/P group did not. Although “pain on walking” did not improve in the WBVT/MA group (mean 28.3 pre-trial to 28.3 post-trial), it actually worsened in the WBVT/P group (mean 26.9 to 24.2). In addition, “pain on ascending or descending stairs” (mean 16.7 to 15.3) also worsened in the WBVT/P group. It appears that a resistance exercise such as WBVT, without the addition of an anti-inflammatory such as MA, is burdensome for knees with severe K-L grades (K-L ≥ 3). Resistance exercise can cause excessive strain on osteoarthritic knees which may lead to further injury or damage [29].

MA functions as an anti-inflammatory and anti-arthritic agent, which is demonstrated by the suppression of carrageenan-induced paw edema, inflammatory cells and destruction of synovium in knee joints [30]. Fukumitsu et al. [31] performed a comparison study of serum hs-CRP between MA and placebo over a 12-week treatment period. The MA intake group had a higher serum hs-CRP level than the placebo group. The authors found that MA improved joint condition and QoL by reducing pain and the inflammatory response. Our results also showed that MA is an effective supplement for participants with severe knee OA (K-L ≥ 3). Decreased knee inflammation alleviated knee pain in participants with severe OA (K-L ≥ 3) in the WBVT/MA group. If MA did not relieve knee inflammation, the knee pain in participants with severe OA would not have improved because it is the severe inflammation that causes the swelling, pain and restricted range of motion in the joint. It is clear that MA intake was effective in relieving knee inflammation for participants with synovitis or joint effusion (K-L ≥ 3). Furthermore, with the gradual reduction in knee inflammation, participants in the WBVT/MA group with K-L grades ≥ 3 could bend their knees with less pain and improved range of motion. They could then fully engage their leg muscles during the WBVT program and build more muscle mass. The “range of motion” domain is directly related to bending the knees.

**Fig 2:**
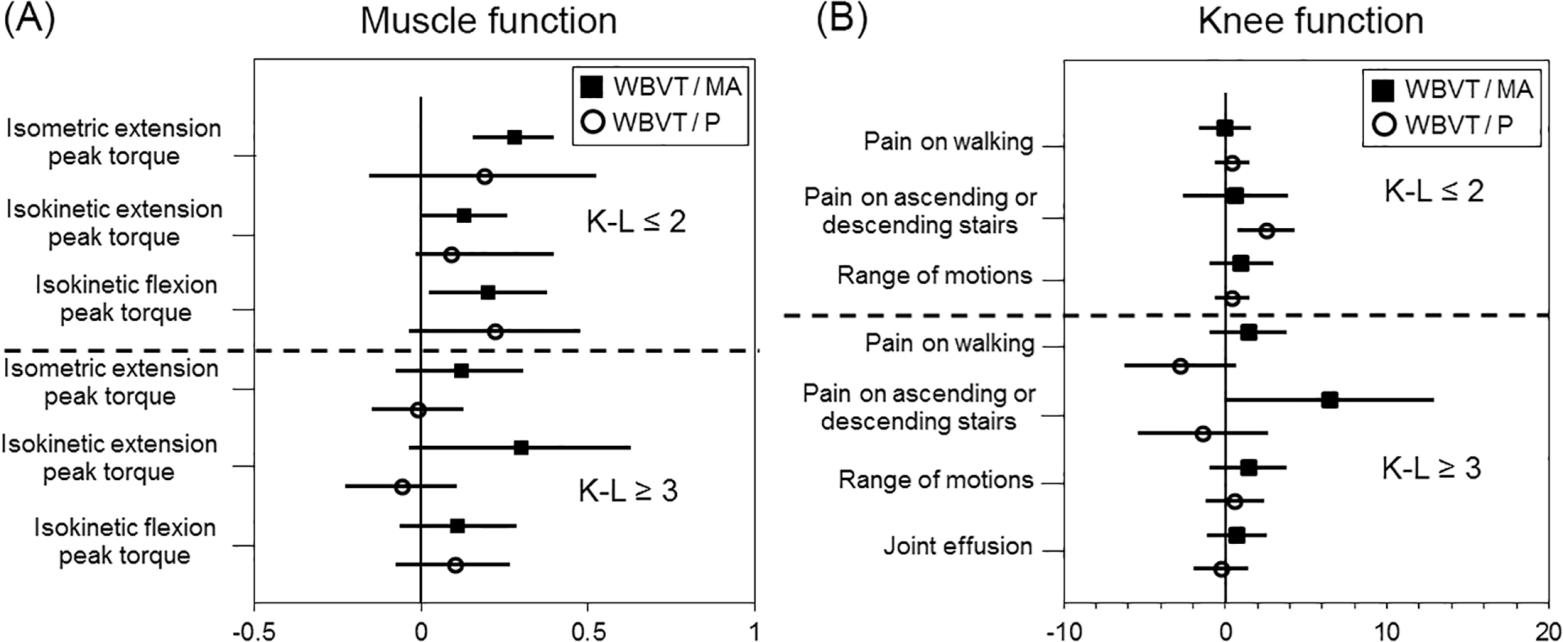
Mean value of the difference between post- and pre-trial for each group (group factor: WBVT/MA and WBVT/P). (A) Muscle function and (B) Knee function. The bars represent upper and lower 95% confidence interval value.

Although, the widely used WOMAC (Western Ontario and McMaster Universities Osteoarthritis Index) [32] is just a questionnaire for participants, JOA score parameters are based on an orthopedist’s interview and diagnosis. The “pain on ascending or descending stairs” and “pain on walking” scores come from interviews, but “range of motion” and “joint effusion” are obtained through clinical assessment. Thus, the JOA score is a quantitative evaluation of physical impairment and disability in patients with knee OA [33]. The JOA “range of motion” domain asks a subject about the “flexion or arc of motion” at angles ranging from 110° to 35°. These angles include the positioning of seiza-style sitting and also evaluates knee pain. For our study, we adopted the JOA score to evaluate the efficacy of WBVT and MA supplementation on elderly women’s knee function. Our results indicated that “pain on walking” and “range of motion” were strongly related to “isokinetic extension peak torque” measurements of the muscle function results. Fig 2 is a visual representation showing the differences in muscle and knee function between the WBVT/MA and WBVT/P groups. This study suggests that, to reduce knee OA in elderly women, the use of MA as an inflammatory drug can be an effective treatment to improve knee muscle strength when used with resistance exercises such as WBVT.

The results do not prove the effect of MA alone in reducing the inflammation since there were a limited number of participants in this study. Therefore, we cannot generalize these findings as a curative and rehabilitative treatment based on this study alone. Furthermore, the potential for response bias as to the efficacy of WBVT/MA must be taken into consideration and tested through the measurement of inflammatory biomarkers such as cytokines.

## Conclusion

An ideal treatment for elderly women with knee OA is a combination of an effective anti-inflammatory supplement, rather than a drug, to reduce knee pain and a low-burden resistance exercise to improve knee muscle strength. Participants in the WBVT/MA group with severe OA (K-L ≥ 3) and synovitis or joint effusion showed remarkable improvement in knee and muscle function during their 20-week training period. This study suggests that to reduce knee pain in elderly women, the anti-inflammatory supplement MA is an effective treatment when used in combination with WBVT for improving knee muscle strength.
